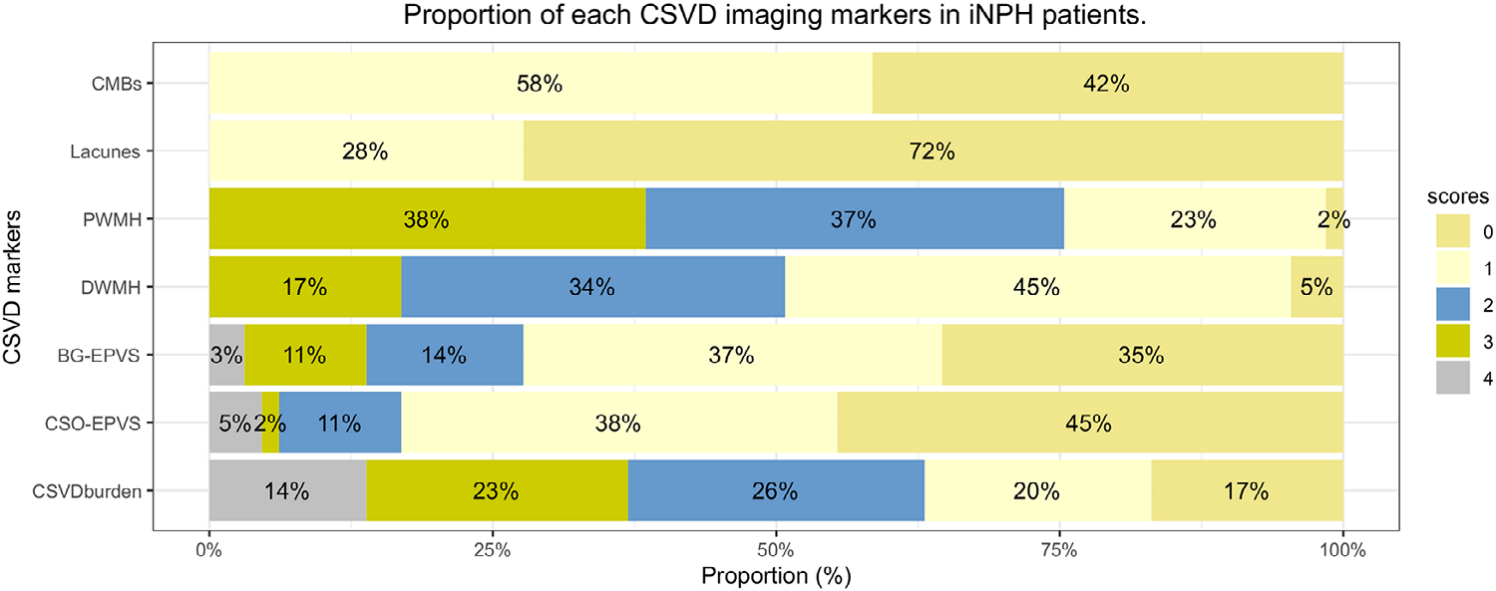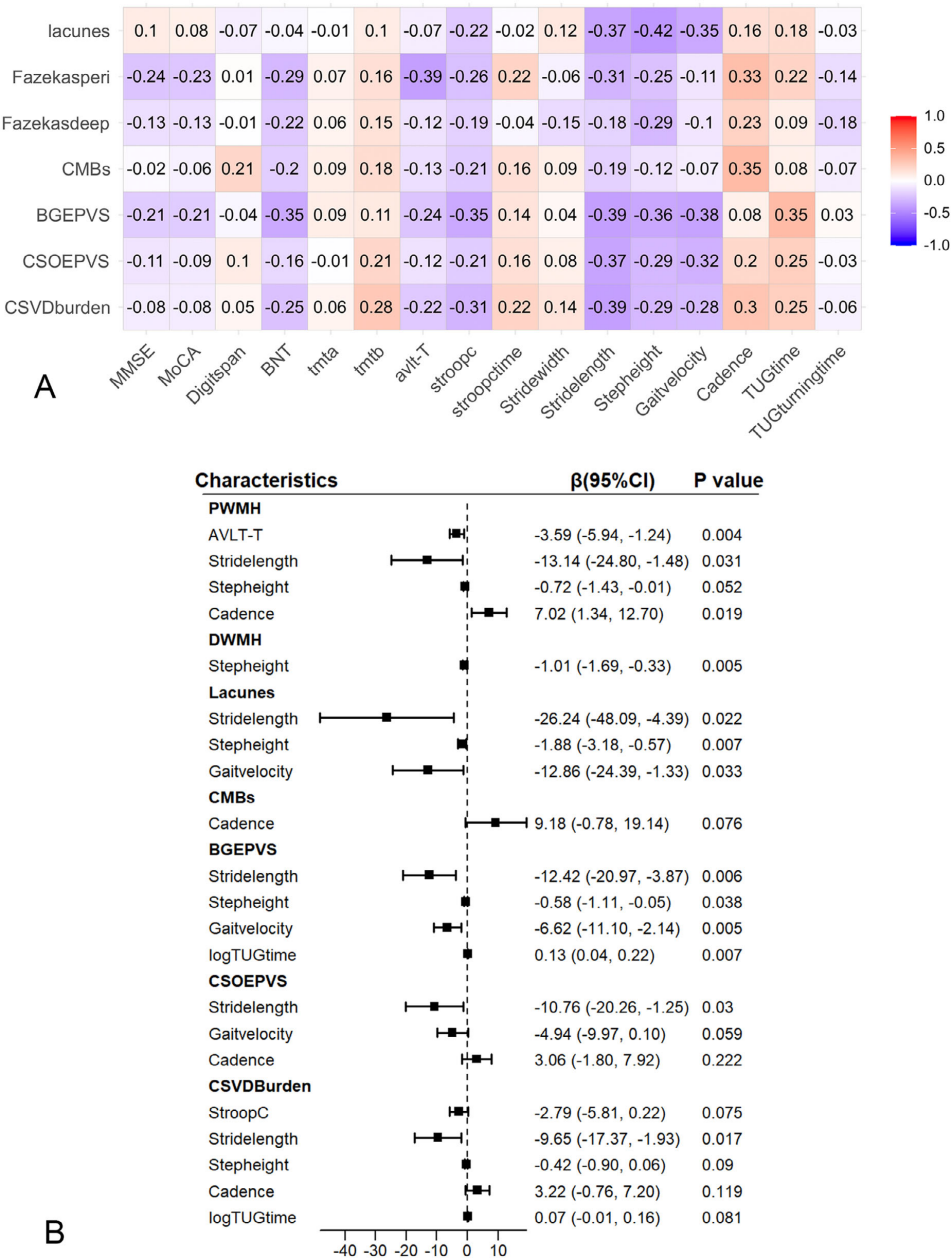# The Role of Cerebral Small Vessel Disease Burden in Idiopathic Normal Pressure Hydrocephalus: Insights from a Prospective Cohort Study

**DOI:** 10.1002/alz.088112

**Published:** 2025-01-09

**Authors:** Hanlin Cai, Keru Huang, Feng Yang, Na Hu, Jiaojiang He, Hui Gao, Shiyu Feng, Linyuan Qin, Ruihan Wang, Shan Wang, Qian Liao, Yi Liu, Liangxue Zhou, Dong Zhou, Zilong Hao, Qin Chen

**Affiliations:** ^1^ West China Hospital of Sichuan University, Chengdu China

## Abstract

**Background:**

The pathogenesis and pathophysiology of idiopathic normal pressure hydrocephalus (iNPH) remain unclear. The cerebral small vessel disease (CSVD) pathology may play a crucial role in patients with iNPH. This study aims to investigate the prevalence of CSVD in patients with probable iNPH and examined the associations between CSVD burden and clinical characteristics and prognosis after shunt surgery.

**Method:**

We enrolled patients with iNPH from an ongoing prospective cohort study in the West China Hospital of Sichuan University from August 2021 to present. The clinical characteristics, cognitive assessment, quantitative gait analyses, and MRI were prospective collected. Those patients who underwent shunt surgery were followed up at 3 months after. CSVD imaging markers including the CSVD burden, PWMH Fazekas, DWMH Fazekas, BG‐EPVS, and CSO‐EPVS scores, and the presence of lacunes and CMBs were evaluated. The linear and logistic regression models were used to investigate the association between the CSVD imaging markers and clinical features and outcomes in patients with iNPH.

**Result:**

Among the 65 patients with iNPH (Age: 74.82 ± 6.23 years, Male: 69%), 83% (54/65) patients had at least one type of CSVD imaging markers and 63% (41/65) patients had CSVD score≧2. Age and hypertension were the highest risk factors. The CSVD score was associated with poorer Stroop test scores, a shorter stride length, lower step height, a faster cadence, and a longer Timed‐Up and Go test time. Adjusting for age, sex, years of education, and vascular risk factors, it remained associated with a shorter stride length. The CSVD score had increased trends for poor improvement in patients with shunt surgery.

**Conclusion:**

Our study showed the high occurrence of CSVD imaging abnormality in patients with iNPH, and the comorbid CSVD were associated with more severe gait disturbances and cognitive decline, suggesting that CSVD may be involved in the pathogenesis of iNPH.